# The power of emojis: The impact of a leader’s use of positive emojis on members’ creativity during computer-mediated communications

**DOI:** 10.1371/journal.pone.0285368

**Published:** 2023-05-18

**Authors:** Jungmin Choi, So-Hyeon Shim, Sara Kim

**Affiliations:** 1 Department of Management, Hong Kong University of Science & Technology, Clear Water Bay, Kowloon, Hong Kong; 2 HKU Business School, The University of Hong Kong, Pokfulam, Hong Kong; Universitat Autònoma de Barcelona: Universitat Autonoma de Barcelona, SPAIN

## Abstract

The existing literature on emojis offers limited insights on the effects of using emojis in organizational settings, especially in the context of leader–member relationships. The current research examines how a leader’s use of positive emojis can influence members’ creative performance, a critical determinant of an organization’s success and productivity. We find that a leader’s use of positive emojis enhances members’ creativity and that this effect is mediated by a decrease in members’ perception of objectification by the leader. We further find that this impact of a leader’s use of positive emojis on members’ creativity is stronger when members have a higher level of relationship orientation. Contrary to the popular belief that the use of emojis in a work setting is inappropriate, our findings reveal that leaders’ use of emojis has positive impacts on important workplace outcomes. These findings provide important guidelines on how to apply emojis to computer-mediated communications at work by demonstrating the circumstances in which positive consequences of using emojis occur.

## Introduction

With the exponential growth of information technology and the digital revolution, computer-mediated communications have become ubiquitous in today’s workplace. Scholars and business practitioners have highlighted that employees in many industries increasingly prefer using e-mail or instant messengers to communicate with their colleagues, clients, and customers [1–3]. This preference for computer-mediated communications has become particularly pronounced since the outbreak of the COVID-19 pandemic, which forced many companies to encourage employees to work from home [4].

One critical and prevalent component of computer-mediated communication is emojis–small digital images or icons used to represent various emotions or animate beings and objects [5]. Emojis are considered an evolution of emoticons, or visual cues formed from textual symbols that represent one’s emotions [6]. Past research has argued that visual cues such as emoticons and emojis play a significant role in computer-mediated communication, as they can convey the senders’ thoughts, emotions, and intentions [7–9]. Because of this communicative function, emojis have become increasingly prevalent in professional settings over time. For instance, according to a survey conducted by Kelton Global of 1,000 American workers with smartphones, 76% of employees in the U.S. use emoticons in computer-mediated conversations with colleagues and clients [3, 10]. Moreover, a recent global survey by technology companies Slack and Duolingo on 9,400 hybrid workers showed that 58% of the respondents believed emojis facilitate communication at work [11].

Despite the prevalence of these visual cues in today’s workplace, the extant literature provides limited insights into the effects of using emojis in organizational settings, especially in the context of leader–member relationships. In fact, prior research implies that the use of emoticons and emojis by leaders could spark members’ negative perceptions of such leaders [12–14]. For example, if lawyers use emoticons in communications with their clients, the latter may perceive the lawyers as unprofessional and overly emotional [13]. Moreover, the use of emojis can lead to perceptions of unprofessionalism or incompetence about the sender in customer-employee relationships [14] as well as in peer relationships [12]. However, it is not clear whether emojis result in such negative perceptions in the context where a leader assigns work to his or her members. As recipients can perceive emojis differently depending on where they are positioned in the organizational hierarchy [15, 16], it is crucial to investigate the impacts of emojis when they are sent by those in a higher position than the recipients. We also examine a particular context wherein a creative task is assigned to a member.

That is, the current research examines situations in which a leader assigns a creative task to a member using positive emojis during computer-mediated conversations. Specifically, we propose that a leader’s use of positive emojis enhances the target member’s creative performance. We further propose that the positive impact of a leader’s use of emojis is mediated by a decrease in members’ perception of being objectified by the leader and that this effect is stronger for members who are more relationship-oriented.

## Theory and hypotheses

### Emojis as nonverbal cues

The word *emoji* comes from two Japanese characters meaning “picture” [e] and “character” [moji]. Emojis surfaced in Japan in 1999 to facilitate online communication [8]. These icons not only include graphical representations of facial expressions but also represent both inanimate objects and living beings. They are often considered a more advanced version of emoticons, which are textual representations of human facial expressions such as “:-)” and “:-(” [5]. Emojis have been observed to progressively replace the role of emoticons in computer-mediated communications [17]. Although emojis are often used and interpreted the same way emoticons are [18, 19], neurological evidence suggests that emojis are more likely to be perceived as human faces than emoticons [20–22]. Therefore, in the current research, we use the term emojis. Moreover, of the various emojis, positive emojis, such as smiling faces and thumbs up, are used more frequently in professional situations [23–26].

Research in the communications and service industries has long recognized the importance of emoji use in computer-mediated communications, as emojis can be nonverbal cues that reveal senders’ facial expressions, emotions, or intentions. Just like nonverbal behaviors in face-to-face communications, emojis offer useful information that complements verbal communication, especially in the context of interpersonal relations [10, 27–29]. This line of research further suggests that the use of emojis can produce positive effects in non-professional contexts. For example, individuals who receive an e-mail using smiley-face emoticons are more likely to perceive the sender as likable than those who receive an e-mail without any smiley-face emoticons [1]. In a related vein, studies have found that e-mail recipients perceive people who send emojis to be more agreeable [30, 31], more sociable [32], and warmer [33]. In addition, emojis can provide additional social information in digital communications [34] by increasing the general clarity of the message communicated by the sender [35], relieving any tensions that may exist between the communicator and the recipient [36], and reducing the recipient’s perception that the sender is taking advantage of them [37].

In contrast to these findings of the positive effects of emoji use in non-professional settings, some scholars and practitioners suggest that emojis used in a professional context may decrease the perceived professionalism of the sender [38]. Guidelines in news articles on computer-mediated communications, or “netiquette,” advise people to limit their use of emojis in workplace communications because they may be perceived as being overly casual and informal and thus, unprofessional [13]. Similarly, scholarly works have suggested that emojis may defy customers’ expectations of formality and create negative perceptions of the sender’s professionalism [39–41].

In the midst of the conflicting findings on emoji use, we believe there are three limitations in the literature on emojis. First, the findings between non-professional and professional settings are inconsistent. Second, although previous studies have examined the impact of emojis during computer-mediated conversations, these studies have focused primarily on recipients’ evaluations regarding the messages and their senders, neglecting to explore the perceptual and behavioral consequences of emojis in organizational contexts, such as how they affect employees psychologically and behaviorally in the workplace, especially in the relationship between leaders and members. Third, while prior work has implied that leaders’ use of emojis in computer-mediated communication at work could have negative effects by signaling a leader’s unprofessionalism, scholars have paid insufficient attention to the conditions under which a leader’s positive emojis can potentially produce positive impacts in the leader–follower relationship in a professional work setting.

In the current research, we introduce a leader’s use of emojis as a theoretical component in the research on leadership and creativity. The goal of the current study is to investigate how positive emojis from a leader influence members’ creativity. Drawing on the literature on nonverbal behaviors and creativity, we propose that when a leader assigns a creative task to a member using positive emojis during a computer-mediated conversation, the member’s creative performance will increase.

### Impacts of a leader’s positive emojis on member creativity

Creativity, or the generation of novel and useful ideas or solutions for processes and products, is a crucial component of organizational productivity and success [6, 42, 43]; for that reason, organizations highly encourage it among their employees. Prior research has shown that leaders play an important role in promoting creativity in various ways, such as through transformational leadership [44], high-quality leader–member exchange [45], and leaders’ emotional intelligence [46].

Studies on the positive impact of leader-related variables on employee creativity emphasize the importance of how leaders communicate with their members. For instance, research on transformational leadership suggests that leaders can enhance creativity among employees through encouragement and a clear vision of given goals [47]. Moreover, increasing the quality of leader–member communication through a high level of leader–member exchange and supportive communication styles of emotionally intelligent leaders contributes to increased creativity among members [48, 49]. These findings consistently suggest that the ways leaders communicate with their members influence the members’ creative performance. Therefore, we focus on whether leaders’ use of emojis, a critical component of computer-mediated communications at work, promotes member creativity.

We argue that a leader’s use of positive emojis while assigning work to members during computer-mediated communications can increase members’ creative performance. We further argue that it is because the positive emojis leaders use may decrease the members’ perceived objectification in the workplace. Objectification is a common psychological phenomenon in the workplace [50]. Objectification occurs when one regards and treats another individual “as more like an object and less like a human being (p.182) [51].” When people feel objectified, they perceive that they are seen as an object with an “instrumental utility” rather than as a human being [51, 52].

Literature on communication and nonverbal behavior supports our proposition that the positive emojis leaders use when they assign work to members during computer-mediated communications may decrease the members’ perceived objectification. A fundamental function of positive nonverbal cues (e.g., a smile, a benign gaze) is to signal the level and quality of a sender’s attention and approval [53, 54]. For example, when individuals receive positive nonverbal attention via an eye gaze from someone during conversation, they believe that person may be interested in interacting with them, accept them, or even value them [53–55]. Considering that emojis represent nonverbal behaviors in computer-mediated communications [56], when members receive positive emojis (e.g., a smiley face) from a leader during computer-mediated communications, they are likely to believe that leader has positive expectations of them or values them. This proposition is also supported by studies on emojis that show that reward-related areas in brain are activated by the presence of emojis [20, 21, 57]. Accordingly, the leader’s positive emojis toward members may be viewed as conveying the leader’s approval or attention, which can potentially lower members’ feeling of objectification (i.e., they are less likely to feel the leader is treating them like an object).

Prior work also provides support for the link between felt objectification and creativity. Past studies on objectification have shown that the cognitive performance of individuals subject to objectification is lower than that of those who are not subject to objectification [58]. For example, a recent study on objectification in an organizational setting found that individuals who were treated as instruments, and who consequently engaged in self-objectification, performed worse in an online work activity than did those who were not treated as instruments [59]. These findings suggest that decreased objectification can improve one’s cognitive performance. One form of cognitive performance that is highly important in today’s organizations is creativity, so this line of studies implies that felt objectification can lower creativity.

More support for the link between felt objectification and creativity comes from the literature on self-surveillance. This stream of research suggests that individuals who perceive a higher level of objectification may be more likely to engage in constant self-surveillance. People with experiences of objectification in interpersonal interactions are likely to internalize the objectifying gaze and engage in greater surveillance of their own actions [58, 60]. Such engagement may hinder the objectified individuals’ creative performance, as creativity generally requires one to “think outside the box (p.82) [61].” Moreover, literature on creativity suggests that persistence is a vital component to improve creativity [62–65]. Indeed, Baas and his colleagues noted that creativity can be enhanced “through hard work, perseverance, and more or less deliberate, persistent, and in-depth exploration of a few cognitive categories or perspectives (p. 740) [66].” As feelings of objectification deplete one’s attentional resources [67], they can hinder persistence in the given task, lowering creativity. That is, members who feel less objectified and thus have the mental capacity to engage in creativity-related tasks over time are likely to generate novel ideas and solutions and demonstrate creative outputs through persistence. Based on the above reasoning, we propose the following hypotheses:

*Hypothesis 1*: *Positive emojis leaders use when assigning work to members during computer-mediated communications increase the members’ creative performance*.
*Hypothesis 2*: *Decreased perceived objectification mediates the relationship between a leader’s use of positive emojis and member creativity*.

### The moderating role of members’ relationship orientation

Further, we propose that the impact of a leader’s positive emojis on member creativity will be affected by the member’s relationship orientation, or the extent to which an individual is concerned about and reacts to relationships with other people. Individuals with a high level of relationship orientation tend to be sensitive to other people’s behaviors [68] and want to form positive relationships with them [69].

Given these characteristics of individuals who are relationship-oriented, we expect that they will be more likely to be affected by a leader’s use of positive emojis. Since relationship-oriented individuals pay a high level of attention to other people’s actions, it is likely that these individuals will be better at noticing a leader’s use of positive emojis, which is a form of nonverbal behavior in computer-mediated communications [56]. Moreover, since individuals with a high level of relationship orientation may strive to form positive relationships with others, they will be more likely than those with a low level of relationship orientation to value a leader’s positive emojis, as the use of positive emojis may signal that the sender is willing to maintain a positive relationship with the receiver [53–55]. Given such appreciation of positive emojis from a leader, relationship-oriented individuals may be more likely to experience a sense of respect and acceptance after receiving these emojis, thereby decreasing perceived objectification by the leader and consequently increasing members’ creative performance. Thus, we argue that:

*Hypothesis 3*: *The indirect effect of a leader’s positive emojis on member creativity through perceived objectification is moderated by the member’s relationship orientation; thus*, *the indirect path is stronger when the member has a high level of relationship orientation*.

## Study overview

**Fig 1:**
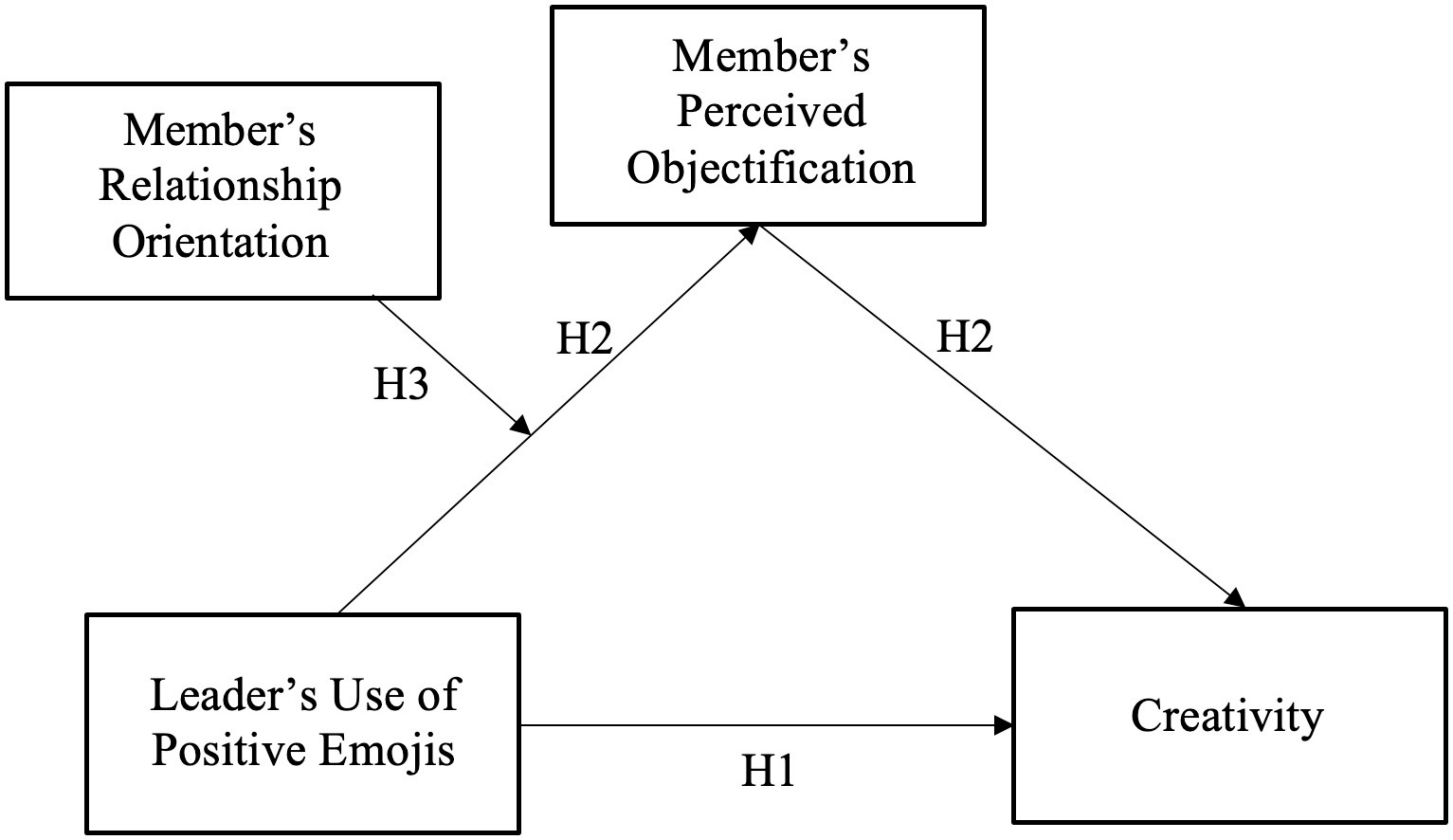
Theoretical model and hypotheses.

Fig 1 depicts our conceptual model. To test the hypotheses, we conducted two studies with experimental designs. In Study 1, we conducted a laboratory experiment to test our primary causal hypothesis–that leaders’ use of positive emojis would influence a member’s creative performance–in the context of real-time computer-mediated communication. In Study 2, we conducted an online experiment to extend the results of Study 1 with three main objectives. The first was to replicate our prediction about the impact of a leader’s use of positive emoji on member creativity. The second objective of Study 2 was to examine perceived objectification as a mechanism underlying the effect of leaders’ use of positive emojis on member creativity through perceived objectification. The final objective of Study 3 was to test the moderating role of members’ relationship orientation in this effect.

To generalize our findings across different types of creativity tasks, Study 1 used an established measure of convergent creativity, which requires one correct response, while Study 2 used a measure of divergent creativity, which requires the generation of multiple ideas [70]. All studies presented in the paper were approved by the Human Research Ethics Committee at the second and third authors’ institution. Data for all studies are available on the Open Science Framework: https://osf.io/qn29h/?view_only=9e2bf922361b496986053790865c4802.

## Study 1 method

### Sample

A total of 159 undergraduate students from a large university in Hong Kong were recruited to participate in the study in exchange for monetary compensation of HK$40 (approximately US$5.1) each. After the exclusion of eight participants who failed the manipulation check and three who did not complete all parts of the experiment, 148 participants remained in the final sample (48 male, 99 female, one non-binary; 96.6% Asian, 1.4% White,.7% Hispanic,.7% Black,.7% Multiracial; China: 87.2%, Other Asian countries: 11.5%, Other Countries: 1.4%; age: *M* = 20.76 years, *SD* = 2.31). Of the participants, 82.4% had previous work experience.

### Procedure

Five confederates were trained to play a role of a leader. They were asked to follow the identical script (e.g., taking the same amount of time to type the same sentence; not adding any additional instructions). We conducted an experiment with one participant at a time, and each participant was paired with one leader. First, each participant was seated in an individual room with a computer. After the participants provided written informed consent to participate in the study, an experimenter told them a leader would designate a task to them via instant messages in an online chat link on the computer assigned to them. They were told that the leader was an experienced researcher at their institution. After the participants were informed about the task, a leader in a different room gave instructions about the Remote Association Task (RAT) [71, 72] through real-time online communication. Specifically, participants were instructed that they would complete a task containing 15 questions; each question in the task would present three unrelated words, and participants would have to think of a fourth word related to the other three words (e.g., bar, dress, and glass; the correct response was *cocktail*) within 30 seconds. Participants were randomly assigned to one of two conditions. In the Emoji condition, the leader provided instructions with positive emojis (i.e., smiley faces and thumbs up). In the Control condition, the leader provided the same instructions but without any emojis (see Supporting information for the stimuli used for each condition). According to the emoji sentiment ranking, the emojis used in the experiment are perceived generally positive [5] (see Supporting information for the details). We ensured that participants engaged in two-way communication with the leader in the following two ways. First, after providing a brief overview of the RAT, the leader asked a practice RAT question, for which participants had to provide a response. Second, at the end of the instructions, participants had the opportunity to ask the leader questions about the task. Participants completed a computerized version of the RAT through an online survey link they received from the leader. After finishing the RAT task, they were told that the task of interacting with the leader was done, and they were asked to complete a short survey containing a manipulation check and questions about their demographic information.

### Measures

#### Manipulation check

To check whether our manipulation was successful, we asked participants to indicate whether they remembered seeing any emojis in the leader instructions (as measured on a binary scale).

#### Creativity

We used a 15-item RAT measure adopted from [72] for member creativity. The RAT is an established measure of creativity [73–75]. The number of questions participants answered correctly served as the dependent variable. Results from a pilot study with 34 participants showed that the questions varied in terms of difficulty. See Supporting information for the results from the pilot study and the list of the fifteen items used in the study.

## Study 1 results

### Manipulation check

Of the participants who completed all parts in the experiment, 91.1% of the participants in the Emoji condition reported that they received emojis from the leader, and 98.7% of the participants in the Control condition reported that did not receive any emojis from the leader, indicating our manipulation of emojis was successful. Participants in the Emoji condition who reported that they did not receive any emojis from the leader and the participant in the Control condition who reported that he or she received emojis from the leader were excluded from all the analyses.

### Creativity

Hypothesis 1 proposes the positive effect of the leader’s use of positive emojis on members’ creativity. To test for this primary hypothesis, we conducted a one-way ANOVA with Condition (0 = Control, 1 = Emoji) as the independent variable and creativity (i.e., performance on the RAT) as the dependent variable. The results revealed that participants in the Emoji condition (*M* = 5.63; *SD* = 3.19) scored higher in terms of RAT performance than did those in the Control condition (*M* = 4.54; *SD* = 2.91), *F*(1, 146) = 4.69, *p* = .032, *η*_p_^2^ = .031, supporting Hypothesis 1. The positive effect of the leader’s use of emojis on the participants’ creativity performance was still significant when we controlled for who was the leader among five confederates (p = .033) and for participants’ age (p = .034), gender (p = .033), ethnicity (p = .030), and previous work experience (p = .025). Results are available in Supporting Information.

## Study 2 method

### Sample

We recruited a total of 201 U.S. participants from Amazon Mechanical Turk through an online survey platform, CloudResearch.com [76]. Of these 201 participants, we excluded 14 who provided an irrelevant response to a given task, seven who failed the attention check, and 29 who failed the manipulation check. Thus, 160 participants remained in the final sample (84 male, 73 female, three non-binary; 76.9% White, 8.1% Black, 8% Multiracial, 6.9% Hispanic, 3.1% Asian). The age distribution of the participants was as follows; 18–24 years: 6.9%; 25–34 years: 38.1%; 35–44 years: 28.1%; 45–54 years: 17.5%; 55–64 years: 7.5%; 65 or older: 1.9%. All participants were compensated US$1.50 for completing the survey.

### Procedure

After providing written informed consent to the survey, the participants were asked to imagine they were employees at an advertising agency. They then saw images of an instant message conversation wherein the leader of their team gave instructions about a new task. As in Study 1, participants were randomly assigned to one of two conditions. In the Emoji condition, the leader provided instructions with positive emojis. For consistency, the emojis used in Study 2 were identical to those used in Study 1. In the Control condition, the leader provided the same instructions without any emojis (the stimuli used for each condition are available in Supporting Information). After receiving the instructions, the participants completed the given task, which was to generate slogans for a new advertisement of a product called “Veggie Burger Meal.” They were asked to generate as many slogans as possible. Following the task, participants completed a questionnaire to assess their perceived objectification by the leader and their relationship orientation. Lastly, they completed manipulation checks and provided their demographic information.

### Measures

#### Manipulation check

Consistent with Study 1, we asked participants to report whether they remembered seeing any emojis during the experiment as a manipulation check.

#### Perceived objectification by the leader

The participants indicated the extent to which they would feel objectified if the instant messages were from their boss in real life using a 7-point scale (from 1 = Strongly Disagree to 7 = Strongly Agree). We adapted four items (α = .94) from an existing measure on dehumanization [77]. The sample items include: “I would feel like the boss doesn’t see me as an individual” and “I would feel like the boss is treating me as if I were an object.”

#### Relationship orientation

To measure the participants’ relationship orientation, they answered a three-item scale adapted from [78] (α = .835) using a 7-point scale ranging from 1 (Strongly Disagree) to 7 (Strongly Agree). The items are: “People are not important for my personal happiness (r),” “It is important for me to be able to get along with other people,” and “My need for people is quite low (r).”

#### Creativity

Consistent with the extant literature on creativity, we defined a creative idea as an idea that is both novel and useful [79, 80]. Based on this definition, we constructed our creativity measure through a procedure established by prior works on creativity [81, 82]. First, we recruited two independent coders who were blind to the study hypotheses to assess the advertisement slogans generated by the participants. The coders were undergraduate research assistants who were fluent in English. After seeing each slogan, the coders provided a separate rating for novelty and usefulness using a scale adapted from [83]. For novelty, the coders indicated the extent to which the “slogan provides a new way to advertise Veggie Burger Meal.” For usefulness, they indicated the extent to which the “slogan is useful.” Both novelty and usefulness were assessed on a 7-point scale ranging from 1 (Strongly Disagree) to 7 (Strongly Agree). As the coders’ ratings for both novelty (ICC1 = .38, ICC2 = .57, *p* < .001; mean *r*_*wg*_ = .82) and usefulness (ICC1 = .37, ICC2 = .57, *p* < .001; mean *r*_*wg*_ = .78) demonstrated acceptable inter-rater reliabilities [84, 85], we constructed the final creativity measure “by multiplying the novelty and usefulness values for each idea and then averaging these scores across all ideas for each participant (p.1788) [82]”.

## Study 2 results

Table 1 presents the means, standard deviations, and correlations.

**Table 1:** Descriptive statistics.

Variable	Mean	*SD*	1. Perceived Objectification	2. Relationship Orientation
1. Perceived Objectification	2.63	1.47		
2. Relationship Orientation	3.72	1.06	-.23**	
3. Creativity	9.02	4.05	-.21**	.06

^†^ *p* < .10;

* *p* < .05;

** *p* < .01

### Manipulation check

In the Emoji condition, 69.8% of the participants indicated that they received emojis from the leader. In the Control condition, all the participants indicated that did not receive any emojis from the leader. We excluded the participants in the Emoji condition who reported that they did not receive any emojis from the leader from all the analyses. Consistent with Study 1, the majority of the participants in the Emoji condition indicated that they received emojis from the leader, providing support for the successful manipulation of the leader’s use of emojis.

### Creativity

To test for Hypothesis 1 on the main effect of leaders’ use of positive emojis on member creativity, a one-way ANOVA with condition (0 = Control, 1 = Emoji) as the independent variable and creativity score as the dependent variable was conducted. The results showed that participants in the Emoji condition (*M* = 9.92; *SD* = 4.44) generated advertisement slogans that were significantly more creative than the slogans generated by the participants in the Control condition (*M* = 8.40; *SD* = 3.65), *F*(1, 158) = 5.64, *p* = .019, *η*_p_^2^ = .034. Hypothesis 1 was supported. The positive effect of the leader’s use of emojis on the participants’ creativity performance was still significant when we controlled for participant age (p = .018), gender (p = .021), ethnicity (p = .014), and occupation (p = .020). Results are available in Supporting Information.

### Mediation analyses

Hypothesis 2 proposed the indirect effect of a leader’s use of positive emojis on members’ creativity, mediated by perceived objectification by that leader. To test for the indirect effect, we conducted a mediation analysis using PROCESS Model 4 (5,000 bootstrap samples) [86]. The results revealed that there was a significant and positive indirect effect of the leader’s use of positive emojis on members’ creativity through a decrease in perceived objectification by the leader, *B* = .24, *SE* = .17, 90% CI [.01,.56], supporting Hypothesis 2. The positive effect of the leader’s use of emojis on the participants’ creativity performance through decreased perception of objectification was still significant when we controlled for participant age, gender, ethnicity, and occupation. Results are available in the Supporting Information.

### Supplementary analyses

As we argue that the effect of leaders’ use of positive emojis on perceived objectification is dependent upon members’ relationship orientation, we conducted supplemental moderation analyses with PROCESS Model 1 (5,000 bootstrap samples) [86]. Results revealed that the interaction between leaders’ use of positive emojis and participants’ relationship orientation had a significant impact on perceived objectification, *B* = -.61, *SE* = .21, 95% CI [-1.02, -.20]. Results further showed that leaders’ use of positive emojis decreased perceived objectification for individuals with a higher relationship orientation (+1SD), *B* = -1.10, *SE* = .32, 95% CI [-1.73, -.48] but did not affect perceived objectification of those with a lower relationship orientation (-1SD), *B* = .19, *SE* = .31, 95% CI [-.43,.81].

### Moderated mediation analyses

**Fig 2:**
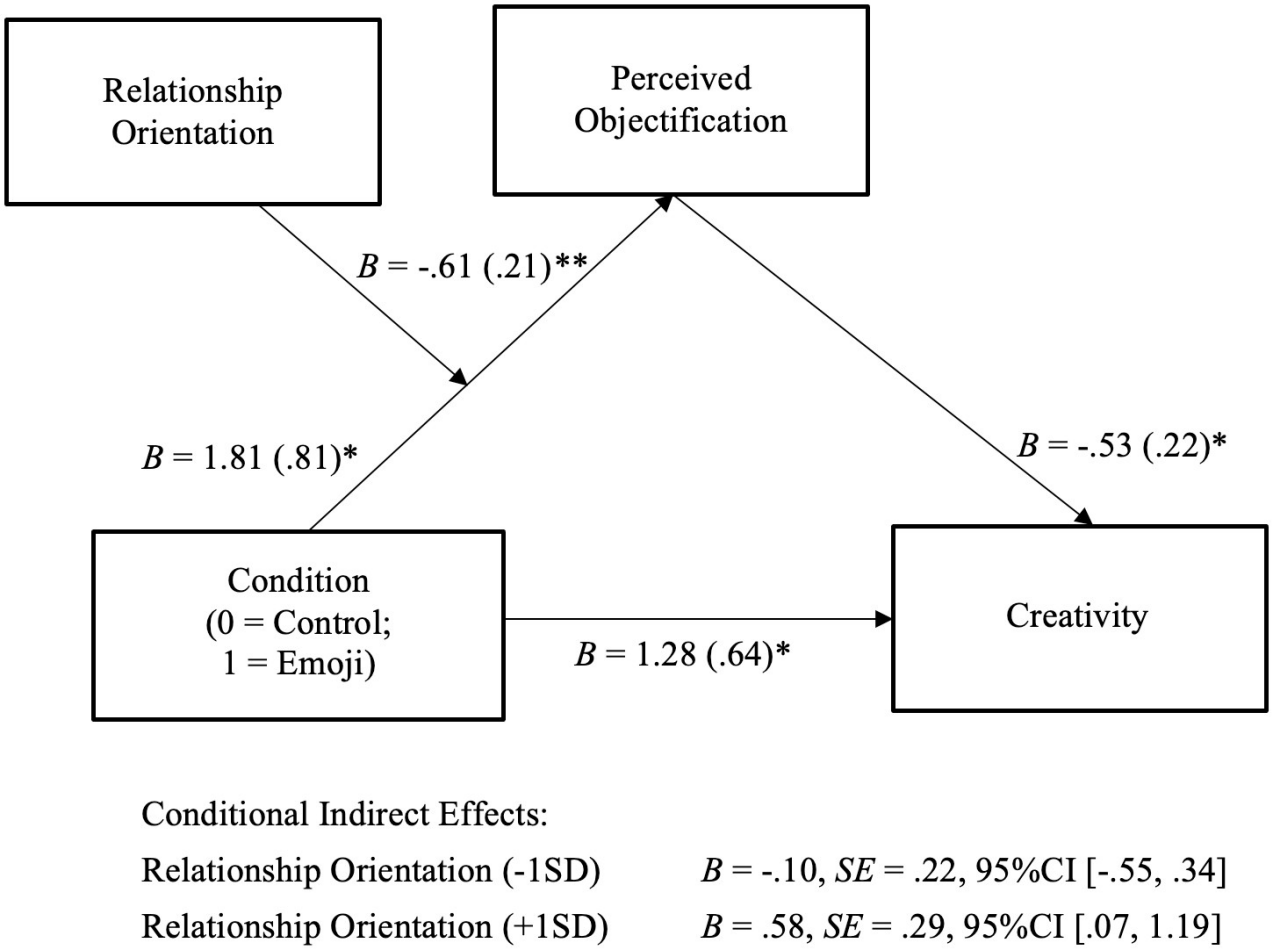
Moderated mediation analyses. †*p <* .10; *p < .05; **p < .01.

To test whether the indirect effect of leader use of positive emojis on member creativity through perceived objectification was moderated by relationship orientation, as proposed by Hypothesis 3, we conducted a moderated mediation analysis using PROCESS Model 7 (5,000 bootstrap samples) [86]. As shown in Fig 2, Condition interacted with relationship orientation to predict creativity through perceived objectification by the leader, *B* = .32, *SE* = .17, 95% CI [.03,.70]. The results revealed that the positive indirect effect of the leader’s use of emojis on creativity through a decrease in perceived objectification by the leader was significant among individuals with a higher level of relationship orientation (+1SD), *B* = .59, *SE* = .28, 95% CI [.09, 1.22], but not among those with a lower level of relationship orientation (-1SD), *B* = -.10, *SE* = .21, 95% CI [-.53,.34]. Based on these findings, Hypothesis 3 was supported. The moderated mediation effect held after controlling for participant age, gender, ethnicity, and occupation. Results are available in the Supporting Information.

## Discussion

Through two experimental studies, we found support for all our hypotheses. First, the leader’s use of positive emojis predicted members’ creativity (Hypothesis 1). This effect was mediated by a decrease in perceived objectification by the leader (Hypotheses 2). In addition, the moderating role of members’ relationship orientation was supported; the positive impact of the leader’s use of emojis was stronger among members with a high level of relationship orientation than those with a low level of such orientation (Hypothesis 3).

### Theoretical contributions

Given the aforementioned findings, the current research makes the following theoretical contributions. First, this research investigated how emojis may lead to both perceptual and behavioral consequences in computer-mediated communication in the workplace. Contrary to existing research, which has focused mainly on the impact of emojis on the receiver’s perception of the sender and the sender’s message [23, 87], the current research provides a greater understanding of how the impact of emojis on a receiver’s perception may translate into a workplace behavior, particularly in the leader–follower relationship.

Second, by introducing a leader’s positive emojis as a theoretical component, this research enhances our understanding of the impact of leaders’ nonverbal behavior on members’ creativity in a computer-mediated communication setting. Previous research on the role of leaders’ behaviors in members’ creative performance has predominantly focused on the effect of different leadership styles, such as transformative [44], authentic [88], and servant leadership [89], on employee creativity, especially in the face-to-face context. While such an approach has enhanced our understanding of the effect of characteristics and behaviors specific to each type of leadership on employee creativity, it has provided a rather narrow view on the direct or overt role of leaders in members’ creative performance. Thus, by studying how leaders’ use of emojis–a common form of subtle and indirect nonverbal behavior in modern virtual workplace setting (such as Zoom)–impacts members’ creativity, this research extends our knowledge on the effects of leaders’ nonverbal behavior on members’ creative performance in a virtual setting.

Third, this research shows that leaders’ use of positive emojis is a factor that influences perceived objectification in the workplace. Although emojis are often viewed as an aid to verbal communication, our findings show they can have a powerful impact in organizations by mitigating objectification, a prevalent phenomenon in the workplace that can produce negative consequences such as low job satisfaction and high turnover [50].

Fourth, some studies suggest that professionals’ use of emojis in computer-mediated communication might produce negative effects, as it can signal that the user is unprofessional or inappropriately emotional in the workplace. In contrast, our study suggests that emojis can bring about positive consequences at work in a leader–member context. We suggest that when a leader uses positive emojis in communication with employees, it can facilitate their creativity, especially when they have a higher level of relationship orientation. These findings enhance our understanding of the positive effects of emojis in the workplace.

Lastly, by examining the influence of perceived objectification and relationship orientation on the relationship between leaders’ use of positive emojis and members’ performance on creative tasks, the current research provides the mechanism behind this relationship and the circumstances under which the relationship occurs.

### Practical implications

The current research provides important implications for managers. Given that creativity is crucial for organizational innovation and growth [90], organizations value and encourage creativity among their employees [91]. To encourage creativity, managers may actively provide positive attention and approval to employees by incorporating positive emojis in computer-mediated communications with them. Results from the two experimental studies showed a positive impact of leaders’ use of positive emojis on both convergent and divergent creativity, suggesting that leaders may use emojis in various organizational situations requiring creativity. The findings from the current research are especially relevant in today’s workplace, as many organizations require their employees to work from home, and virtual communication between employees and managers has become more important than ever.

### Limitations and future directions

The current research is subject to several limitations that suggest directions for future research. First, our research was conducted in a context in which the leader did not have any prior relationship with the member. As this research focused on establishing the baseline effect of leaders’ use of emojis on employee creativity, we did not consider the nature of the leader–member relationship. However, based on the effects found in our research, future studies could investigate whether the characteristics of leader–member relationships moderate the effects. For instance, if a leader uses positive emojis while communicating with a member with whom he or she does not have a positive relationship, the use of emojis might backfire instead of increasing member creativity.

Second, it would be meaningful to examine the role of one’s cultural background in the relationship between leader’s use of emojis and employee creativity. The current research examined the impact of leader’s use of positive emojis on employee creativity in different cultures across two studies. As both studies provide support for the hypotheses, there is support for the generalizability of the positive impact of leader’s use of positive emojis on employee creativity across different cultures. However, future research can explore situations where the impact of leader’s use of emojis might depend on culture. For example, past research on emojis suggest that there may be cultural differences in interpretation of emojis that are not related to happiness [92]. Thus, it would be interesting to explore whether there will be cultural differences in interpreting leader’s use of emojis that are not positive.

In a related vein, it would be interesting to explore the role of other demographic variables such as gender and age. Although our results did not show differences in male and female participants, it would be worthwhile to explore whether the findings from the current research will hold in a mixed-gender leader-employee relationship. Moreover, future research can examine whether the results from the current research will be applicable to a non-traditional relationship where a leader is younger than employees.

Moreover, to gain a more comprehensive understanding of the effect of leaders’ use of emojis, future studies could examine the impact of leaders’ use of negative emojis. Although positive emojis are more commonly used in organizational settings, emojis with a negative valence are also seen in computer-mediated communications at work [26]. Thus, it would be worthwhile to investigate whether negative emojis leaders use have a differential effect on employee creativity compared to positive emojis. In a related vein, future studies could also test whether the effects of emojis differ depending on the number used.

Finally, in our study, we conducted a laboratory experiment and an online experiment to test the hypothesized effects. While conducting the experiments allowed us to test the effects in a controlled manner by minimizing the impacts of potential confounding variables, it would be meaningful to increase the external validity of the findings from the current research via a field study.

## Conclusion

The current research examines the impacts of a leader’s use of positive emojis on members’ creativity. Individuals who receive positive emojis from their leaders may perform more creatively, as they may feel less objectified by their leaders. Our results also suggest that individuals who are more relationship-oriented may perform more creatively than those who are less relationship-oriented. This research starts a useful conversation on the role of emojis, which are a growing component of computer-mediated communications in the organizational context.

## Supporting information

S1 Table Study 1 pilot study results on RAT. (PDF)

S1 Appendix Study 1 stimuli used in each condition. (PDF)

S2 Appendix Information about the emojis used in studies based on emoji sentiment ranking. (PDF)

S3 Appendix Study 1 results controlling for confederate leader, participant age, gender, ethnicity, and previous work experience. (PDF)

S4 Appendix Study 1 results including all participants. (PDF)

S5 Appendix Study 2 stimuli used in each condition. (PDF)

S6 Appendix Study 2 ANOVA results controlling for participant age, gender, ethnicity, and occupation. (PDF)

S7 Appendix Study 2 mediation results controlling for participant age, gender, ethnicity, and occupation. (PDF)

S8 Appendix Study 2 moderated mediation results controlling for participant age, gender, ethnicity, and occupation. (PDF)

S9 Appendix Study 2 results including all participants. (PDF)

## References

[1] Byron K, Baldridge DC. E-mail recipients’ impressions of senders’ likability: The interactive effect of nonverbal cues and recipients’ personality. J Bus Commun. 2007;44(2):137–60. 10.1177/0021943606297902

[2] Baldwin H. Instant messaging is going corporate. Forbes. 2014 [cited 2022 Jun 28]. https://www.forbes.com/sites/howardbaldwin/2014/02/17/instant-messaging-is-going-corporate/

[3] Lee BY. Use emojis in work emails? You may be tainting your colleagues’ opinion of you. Forbes. 2017 [cited 2022 Jun 28]. https://www.forbes.com/sites/brucelee/2017/08/15/using-emojis-at-work-beware-of-this-risk/

[4] Kier L. Virtual communication is vital to business resilience. Forbes. 2020 [cited 2022 Jun 28]. https://www.forbes.com/sites/forbescommunicationscouncil/2020/09/16/virtual-communication-is-vital-to-business-resilience/

[5] Kralj Novak P, Smailović J, Sluban B, Mozetič I. Sentiment of emojis. PLoS One. 2015 10(12): e0144296. doi: 10.1371/journal.pone.0144296 26641093 PMC4671607

[6] Rezabek L, Cochenour J. Visual cues in computer-mediated communication: Supplementing text with emoticons. J Vis Lit. 1998;18(2):201–15. 10.1080/23796529.1998.11674539

[7] Walther JB, D’Addario KP. The impacts of emoticons on message interpretation in computer-mediated communication. Soc Sci Comput Rev. 2001;19(3):324–47. 10.1177/089443930101900307

[8] Bai Q, Dan Q, Mu Z, Yang M. A systematic review of emoji: Current research and future perspectives. Front Psychol. 2019 Oct 15;10:2221. doi: 10.3389/fpsyg.2019.02221 31681068 PMC6803511

[9] Pfeifer VA, Armstrong EL, Lai VT. Do all facial emojis communicate emotion? The impact of facial emojis on perceived sender emotion and text processing. Comput Hum Behav. 2022 Jan 1;126:107016.

[10] Skovholt K, Grønning A, Kankaanranta A. The communicative functions of emoticons in workplace E-mails:: -). J Comput Mediat Commun. 2014;19(4):780–97. 10.1111/jcc4.12063

[11] Slack. Beyond the smile: how emoji use has evolved in the workplace. Slack. 2022 [cited 2023 Feb 25]. https://slack.com/blog/collaboration/emoji-use-at-work

[12] Glikson E, Cheshin A, van Kleef GA. The dark side of a smiley: Effects of smiling emoticons on virtual first impressions. Soc Psychol Personal Sci. 2018;9(5):614–25. 10.1177/1948550617720269

[13] Lebovits G. E-mail netiquette for lawyers. NY State Bar J. 2015;81(9): 56–9.

[14] Li X, Chan KW, Kim S. Service with emoticons: How customers interpret employee use of emoticons in online service encounters. J Consum Res. 2019;45(5):973–87. 10.1093/jcr/ucy016

[15] Lundberg C. Person-focused joking: Pattern and function. Hum Organ. 1969;28(1):22–8. 10.17730/humo.28.1.273176718w578211

[16] Dwyer T. Humor, power, and change in organizations. Hum Relat. 1991;44(1):1–19. 10.1177/001872679104400101

[17] Chairunnisa S, Benedictus AS. Analysis of emoji and emoticon usage in interpersonal communication of Blackberry messenger and WhatsApp application user. Int J of Soc Sci Manag. 2017 Apr 24;4(2):120–6. doi: 10.3126/ijssm.v4i2.17173

[18] Ganster T, Eimler SC, Krämer NC. Same same but different!? The differential influence of smilies and emoticons on person perception. Cyberpsychol Behav Soc Netw. 2012;15(4):226–30. 10.1089/cyber.2011.0179 22394421

[19] Neel LA, McKechnie JG, Robus CM, Hand CJ. Emoji alter the perception of emotion in affectively neutral text messages. J Nonverbal Behav. 2023 Jan 17. doi: 10.1007/s10919-022-00421-6

[20] Chatzichristos C, Morante M, Andreadis N, Kofidis E, Kopsinis Y, Theodoridis S. Emojis influence autobiographical memory retrieval from reading words: An fMRI-based study. PLoS One. 2020 Jul 1;15(7): e0234104. doi: 10.1371/journal.pone.0234104 32609778 PMC7329082

[21] Yuasa M, Saito K, Mukawa N. Brain activity when reading sentences and emoticons: An fMRI study of verbal and nonverbal communication. Electr and Commun in Japan. 2011;94(5):1797–1803. doi: 10.1002/ecj.10311

[22] Yuasa M, Saito K, Mukawa N. Emoticons convey emotions without cognition of faces: An fMRI study. In: Human Factors in Computing Systems (CHI). Que´bec, Canada; 2006.

[23] Wang W, Zhao Y, Qiu L, Zhu Y. Effects of emoticons on the acceptance of negative feedback in computer-mediated communication. J Assoc Inf Syst. 2014;15(8):454–83. 10.17705/1jais.00370

[24] Hancock JT, Dunham PJ. Impression formation in computer-mediated communication revisited: An analysis of the breadth and intensity of impressions. Communic Res. 2001;28(3):325–47. 10.1177/009365001028003004

[25] Wall HJ, Kaye LK, Malone SA. An exploration of psychological factors on emoticon usage and implications for judgement accuracy. Comput Human Behav. 2016;62:70–8. 10.1016/j.chb.2016.03.040

[26] Cultivate. Leadership trends: emoji usage at work. Cultivate. 2020 [cited 2023 Feb 25]. https://cultivate.com/wp-content/uploads/2020/08/Cultivate-Data-Report_Emoji-Usage-at-Work.pdf

[27] Albright L, Kenny DA, Malloy TE. Consensus in personality judgments at zero acquaintance. J Pers Soc Psychol. 1988;55(3):38795. 10.1037//0022-3514.55.3.387 3171912

[28] Gibson W, Huang P, Yu Q. Emoji and communicative action: The semiotics, sequence and gestural actions of ‘face covering hand’. Discourse Context & Media. 2018 Dec 1;26:91–9. doi: 10.1016/j.dcm.2018.05.005

[29] Gesselman AN, Ta VP, Garcia JR. Worth a thousand interpersonal words: Emoji as affective signals for relationship-oriented digital communication. PLoS One. 2019 Aug 15;14(8):e0221297. doi: 10.1371/journal.pone.0221297 31415640 PMC6695182

[30] Fullwood C, Martino OI. Emoticons and impression formation. Early Pop Vis Cult. 2007;19(8): 4–14.

[31] Taesler P, Monique J. Emoticons and impression formation: The impact of emoticon use on the perception of online communication partners. Gruppendynamik und Organisationsberatung. 2007;41(4):375–84.

[32] Zhang L, Lee BE, Heidi CW. 2011. Effects of ‘emotional text’ on online customer service chat,” In: Proceedings of Graduate Student Research Conference in Hospitality and Tourism; 2011; Houston, TX.

[33] Hand CJ, Burd K, Oliver A, Robus CM. Interactions between text content and emoji types determine perceptions of both messages and senders. Comput Hum Behav Reports. 2022 Dec 1;8:100242. doi: 10.1016/j.chbr.2022.100242

[34] Tung F-W, Deng Y-S. Increasing social presence of social actors in e-learning environments: Effects of dynamic and static emoticons on children. Displays. 2007;28(4–5):174–80. 10.1016/j.displa.2007.06.005

[35] Riordan MA. The communicative role of non-face emojis: affect and disambiguation. Comput Hum Behav. 2017 Nov 1;76:75–86. doi: 10.1016/j.chb.2017.07.009

[36] Moye NA, Langfred CW. Information sharing and group conflict: Going beyond decision making to understand the effects of information sharing on group performance. Int J Confl Manag. 2004;15(4):381–410. 10.1108/eb022919

[37] Thompsen PA, Foulger DA. Effects of pictographs and quoting on flaming in electronic mail. Comput Human Behav. 1996;12(2):225–43. 10.1016/0747-5632(96)00004-0

[38] Munter M, Rogers PS, Rymer J. Business E-mail: Guidelines for users. Bus Commun Q. 2003;66(1):26–40. 10.1177/108056990306600104

[39] Ellensburg R. Emoticons as social information: The effect of emoticons on interpersonal perception in computer-mediated business communication [thesis on the Internet]. Amsterdam: University of Amsterdam; 2012 [cited 2022 Jun 28]. https://arno.uva.nl/cgi/arno/show.cgi?fid=463478.

[40] Gacey HJ, Richard EM. Influence of emoticons on perceived negative affect and professionalism in work-related email. Acad Manag Proc. 2013;2013(1):14646. 10.5465/ambpp.2013.14646abstract

[41] Haberstroh S. College counselors’ use of informal language online: student perceptions of expertness, trustworthiness, and attractiveness. Cyberpsychol Behav Soc Netw. 2010;13(4):455–9. 10.1089/cyber.2009.0280 20712504

[42] Zhou J, George JM. When job dissatisfaction leads to creativity: Encouraging the expression of voice. Acad Manage J. 2001;44(4):682–96. 10.2307/3069410

[43] Howell JM. The right stuff: Identifying and developing effective champions of innovation. Acad Manag Perspect. 2005;19(2):108–19. 10.5465/ame.2005.16965104

[44] Shin SJ, Zhou J. Transformational leadership, conservation, and creativity: Evidence from Korea. Acad Manage J. 2003;46(6):703–14. 10.5465/30040662

[45] Tierney P, Farmer SM, Graen GB. An examination of leadership and employee creativity: The relevance of traits and relationships. Pers Psychol. 1999;52(3):591–620. 10.1111/j.1744-6570.1999.tb00173.x

[46] Rego A, Sousa F, Pina e Cunha M, Correia A, Saur-Amaral I. Leader self-reported emotional intelligence and perceived employee creativity: An exploratory study. Creat Innov Manag. 2007;16(3):250–64. 10.1111/j.1467-8691.2007.00435.x

[47] Gumusluoglu L, Ilsev A. Transformational leadership, creativity, and organizational innovation. J Bus Res. 2009 Apr 1;62(4):461–73. doi: 10.1016/j.jbusres.2007.07.032

[48] Atwater L, Carmeli A. Leader–member exchange, feelings of energy, and involvement in creative work. Leadersh Q. 2009 Jun 1;20(3):264–75. doi: 10.1016/j.leaqua.2007.07.009

[49] Zhou J, George JM. Awakening employee creativity: The role of leader emotional intelligence. Leadersh Q. 2003 Aug 1;14(4–5):545–68. doi: 10.1016/S1048-9843(03)00051-1

[50] Belmi P, Schroeder J. Human “resources”? Objectification at work. J Pers Soc Psychol. 2021;120(2):384–417. 10.1037/pspi0000254 32658521

[51] Morris KL, Goldenberg JL, Heflick NA. Trio of terror (pregnancy, menstruation, and breastfeeding): an existential function of literal self-objectification among women. J Pers Soc Psychol. 2014;107(1):181–98. 10.1037/a0036493 24956319

[52] Loughnan S, Baldissarri C, Spaccatini F, Elder L. Internalizing objectification: Objectified individuals see themselves as less warm, competent, moral, and human. Br J Soc Psychol. 2017;56(2):217–32. 10.1111/bjso.12188 28198021

[53] Friedman N. Social nature of psychological research: Psychological experiment as social interaction. La Vergne, TN: Basic Books; 1967.

[54] Wirth JH, Sacco DF, Hugenberg K, Williams KD. Eye gaze as relational evaluation: averted eye gaze leads to feelings of ostracism and relational devaluation. Pers Soc Psychol Bull. 2010;36(7):869–82. 10.1177/0146167210370032 20505162

[55] Jones RA, Cooper J. Mediation of experimenter effects. J Pers Soc Psychol. 1971;20:70–74. doi: 10.1037/h0031700

[56] Derks D, Bos AER, von Grumbkow J. Emoticons and online message interpretation. Soc Sci Comput Rev. 2008;26(3):379–88. 10.1177/0894439307311611

[57] Weiß M, Mussel P, Hewig J. The value of a real face: Differences between affective faces and emojis in neural processing and their social influence on decision-making. Soc Neurosci. 2020;15(3):255–268. doi: 10.1080/17470919.2019.1675758 31581887

[58] Winn L, Cornelius R. Self-objectification and cognitive performance: A systematic review of the literature. Front Psychol. 2020;11:20. 10.3389/fpsyg.2020.00020 32047457 PMC6997128

[59] Baldissarri C, Andrighetto L. Being treated as an instrument: Consequences of instrumental treatment and self-objectification on task engagement and performance. Hum Perform. 2021;34(2):85–106. 10.1080/08959285.2021.1878182

[60] Calogero RM. Objects don’t object: evidence that self-objectification disrupts women’s social activism: Evidence that self-objectification disrupts women’s social activism. Psychol Sci. 2013;24(3):312–8. 10.1177/0956797612452574 23341162

[61] Mumford MD. Handbook of organizational creativity. San Diego, CA: Academic Press; 2018.

[62] Baas M, De Dreu CKW, Nijstad BA. When prevention promotes creativity: the role of mood, regulatory focus, and regulatory closure. J Pers Soc Psychol. 2011;100(5):794–809. 10.1037/a0022981 21381857

[63] Baas M, Koch S, Nijstad BA, De Dreu CKW. Conceiving creativity: The nature and consequences of laypeople’s beliefs about the realization of creativity. Psychol Aesthet Creat Arts. 2015;9(3):340–54. 10.1037/a0039420

[64] Roskes M, De Dreu CKW, Nijstad BA. Necessity is the mother of invention: Avoidance motivation stimulates creativity through cognitive effort. J Pers Soc Psychol. 2012;103(2):242–56. 10.1037/a0028442 22564013

[65] Hennessey BA, Amabile TM. Creativity. In: Fiske S, editor. Annu Rev Psychol. Palo Alto, CA: Annual Reviews;2010. p. 569–98. 10.1146/annurev.psych.093008.100416 19575609

[66] Baas M, De Dreu CKW, Nijstad BA. A meta-analysis of 25 years of mood-creativity research: Hedonic tone, activation, or regulatory focus? Psychol Bull. 2008;134(6):779–806. 10.1037/a0012815 18954157

[67] Fredrickson BL, Roberts T-A. Objectification theory: Toward understanding women’s lived experiences and mental health risks. Psychol Women Q. 1997;21(2):173–206. 10.1111/j.1471-6402.1997.tb00108.x

[68] Swap WC, Rubin JZ. Measurement of interpersonal orientation. J Pers Soc Psychol. 1983;44(1):208–19. 10.1037/0022-3514.44.1.208

[69] Vogt DS, Colvin CR. Interpersonal orientation and the accuracy of personality judgments: Interpersonal orientation and accuracy. J Pers. 2003;71(2):267–95. 10.1111/1467-6494.7102005 12693518

[70] Runco MA. To understand is to create: an epistemological perspective on human nature and personal creativity. In: Richards R, editor. Everyday creativity and new views of human nature: Psychological, social, and spiritual perspectives. American Psychology Association; 2007. p. 91–107.

[71] Mednick SA. Remote associates test. J Creat Behav. 1968;2:213–214.

[72] Bowden EM, Jung-Beeman M. Normative data for 144 compound remote associate problems. Behav Res Meth Instrum Comput. 2003 Nov;35:634–9. doi: 10.3758/bf03195543 14748508

[73] Sassenberg K, Moskowitz GB, Fetterman A, Kessler T. Priming creativity as a strategy to increase creative performance by facilitating the activation and use of remote associations. J Exp Soc Psychol. 2017 Jan 1;68:128–38. doi: 10.1016/j.jesp.2016.06.010

[74] Ritter SM, Ferguson S. Happy creativity: Listening to happy music facilitates divergent thinking. PLoS One. 2017 Sep 6;12(9):e0182210. doi: 10.1371/journal.pone.0182210 28877176 PMC5587106

[75] Zmigrod S, Colzato LS, Hommel B. Stimulating creativity: Modulation of convergent and divergent thinking by transcranial direct current stimulation (tDCS). Creat Res J. 2015 Oct 2;27(4):353–60. doi: 10.1080/10400419.2015.1087280

[76] Litman L, Robinson J, Abberbock T. Turkprime.com: A versatile crowdsourcing data acquisition platform for the behavioral sciences. Behav Res Methods. 2017;49(2):433–42. 10.3758/s13428-016-0727-z 27071389 PMC5405057

[77] Bastian B, Haslam N. Experiencing dehumanization: Cognitive and emotional effects of everyday dehumanization. Basic Appl Soc Psych. 2011;33(4):295–303. 10.1080/01973533.2011.614132

[78] Filsinger EE. A measure of interpersonal orientation: The liking people scale. J Pers Assess. 1981;45: 295–300. doi: 10.1207/s15327752jpa4503_11 16370715

[79] Amabile TM. Creativity in context: Update to “The social psychology of creativity.” Boulder, CO: Westview Press; 1996.

[80] Zhou J, Shalley CE. Deepening our understanding of creativity in the workplace: A review of different approaches to creativity research. In: Zedeck S, editor. APA handbook of industrial and organizational psychology, vol 1. Washington, DC: American Psychological Association;2011. p. 275–302.

[81] Hoever IJ, van Knippenberg D, van Ginkel WP, Barkema HG. Fostering team creativity: Perspective taking as key to unlocking diversity’s potential. J Appl Psychol. 2012;97(5):982–96. 10.1037/a0029159 22774764

[82] Brown G, Baer M. Protecting the turf: The effect of territorial marking on others’ creativity. J Appl Psychol. 2015;100: 1785–97. doi: 10.1037/a0039254 25938721

[83] Lu S, Bartol KM, Venkataramani V, Zheng X, Liu X. Pitching novel ideas to the boss: The interactive effects of employees’ idea enactment and influence tactics on creativity assessment and implementation. Acad Manage J. 2019;62(2):579–606. 10.5465/amj.2016.0942

[84] Bliese PD Within group agreement, non-independence and reliability: Implications for data and analysis. In Klein KJ, Kozlowski SWJ, editors. Multilevel theory, research and methods in organizations: Foundations, extensions, and new directions. San Francisco: Jossey-Bass;2000. p. 349–381.

[85] LeBreton JM, Senter JL. Answers to 20 questions about interrater reliability and interrater agreement. Organ Res Methods. 2008 Oct;11(4):815–52.

[86] Hayes A. Introduction to mediation, moderation, and conditional process analysis: A regression-based approach. New York: Guilford Press; 2013.

[87] Ernst C-P, Huschens M. Friendly, humorous, incompetent? On the influence of emoticons on interpersonal perception in the workplace. In: Proceedings of the 52nd Hawaii International Conference on System Sciences; 2019; Hawaii.

[88] Rego A, Sousa F, Marques C, Cunha MP e. Authentic leadership promoting employees’ psychological capital and creativity. J Bus Res. 2012;65(3):429–37. 10.1016/j.jbusres.2011.10.003

[89] Yoshida DT, Sendjaya S, Hirst G, Cooper B. Does servant leadership foster creativity and innovation? A multi-level mediation study of identification and prototypicality. J Bus Res. 2014;67(7):1395–404. 10.1016/j.jbusres.2013.08.013

[90] Baer M, Oldham GR. The curvilinear relation between experienced creative time pressure and creativity: Moderating effects of openness to experience and support for creativity. J Appl Psychol. 2006;91(4):963–70. 10.1037/0021-9010.91.4.963 16834519

[91] Thompson L. Improving the creativity of organizational work groups. Acad Manag Perspect. 2003;17(1):96–109. 10.5465/ame.2003.9474814

[92] Guntuku S, Li M, Tay L, Ungar L. Studying cultural differences in emoji usage across the East and the West. In: ICWSM 2019: Proceedings of the 13th International AAAI Conference on Web and Social Media; 2019 June 11–14; Münich, Germany. p. 226–35.

