# Supplementary material for: The power of emojis: The impact of a leader’s use of positive emojis on members’ creativity during computer-mediated communications
Source: PLoS One. 2023 May 18;18(5):e0285368. doi: 10.1371/journal.pone.0285368 (PMC10194970; doi:10.1371/journal.pone.0285368)
Supplement: S1 Table — (PDF) [file pone.0285368.s001.pdf]

S3 Table. Study 1 Pilot Study Results on RAT

Question	% of participants solved correctly
cream_skate_water (correct response *ice*)	35.29
dew_comb_bee (correct response *honey*)	26.47
print_berry_bird (correct response *blue*)	23.53
sleeping_bean_trash (correct response *bag*)	20.59
food_forward_break (correct response *fast*)	17.65
cane_daddy_plum (correct response *sugar*)	14.71
cottage_swiss_cake (correct response *cheese*)	8.82
aid_rubber_wagon (correct response *band*)	8.82
down_question_check (correct response *mark*)	8.82
sense_courtesy_place (correct response *common*)	8.82
type_ghost_screen (correct response *writer*)	5.88
opera_hand_dish (correct response *soap*)	5.88
trip_house_goal (correct response *field*)	5.88
wise_work_tower (correct response *clock*)	5.88
