# Supplementary material for: The power of emojis: The impact of a leader’s use of positive emojis on members’ creativity during computer-mediated communications
Source: PLoS One. 2023 May 18;18(5):e0285368. doi: 10.1371/journal.pone.0285368 (PMC10194970; doi:10.1371/journal.pone.0285368)
Supplement: S1 Appendix — (PDF) [file pone.0285368.s002.pdf]

S1 Appendix. Study 1 Stimuli Used in Each Condition

Emoji Condition

Hi [Participant Name]

I am Alex, and I am the leader in this task. 😃

As mentioned by the experimenter, there is a task I would like to assign to you.

Please hear me out until I finish my instructions.

Then, if there is anything that is not clear, you can ask me questions.

This task has 15 questions.

Each question in this task lists three words, and your job is to come up with a fourth word that is related to all three words.

For example, for a question that lists "bar – dress – glass",

the correct fourth word that connects the three words would be "cocktail" (cocktail bar, cocktail dress, cocktail glass).

The answer may not always come before each word in the question. The answer is a word that is "associated with" all three words in a question.

For example, for a question that lists "cat – number – phone",

the correct fourth word is "call" (catcall, call number, phone call).

You will be given 30 seconds to complete each question. After 30 seconds, the page will automatically move to the next question.

It is important that you complete this task with your own ability, so please try your best to come up with the answer on your own. 😊

Before you start the task, let's practice together. I will give 30 seconds to wait for you to type the answer.

Here is the question. What would be the fourth word that connects "broken – clear – eye"?

Please type your response in 30 seconds.

[The leader waits 30 seconds] What is your answer?

[If the answer is correct]: Nice try. 😊 Your answer is correct.
[If the answer is incorrect]: Nice try. 😊 The correct answer is “glass” (broken glass, clear glass, eyeglass).
[If the participant did not provide any answer]: Seems like you needed more time. Nice try. 😊 The correct answer is “glass” (broken glass, clear glass, eyeglass).

Do you have any questions?

[The leader answers the questions]

[If participants do not have further questions]: Great. Let’s start. 👍

Please complete the task in the following link: [Link to the RAT Task]

Let me know in this chat when you are finished with the task.

[After participants lets the leader know that they are finished]: Thanks. Our interaction ends here. I will now tell the experimenter to give you the further instructions.

Control Condition

Hi [Participant Name]

I am Alex, and I am the leader in this task.

As mentioned by the experimenter, there is a task I would like to assign to you.

Please hear me out until I finish my instructions.

Then, if there is anything that is not clear, you can ask me questions.

This task has 15 questions.

Each question in this task lists three words, and your job is to come up with a fourth word that is related to all three words.

For example, for a question that lists “bar – dress – glass”,

the correct fourth word that connects the three words would be “cocktail” (cocktail bar, cocktail dress, cocktail glass).

The answer may not always come before each word in the question. The answer is a word that is “associated with” all three words in a question.

For example, for a question that lists “cat – number – phone”,

the correct fourth word is “call” (catcall, call number, phone call).

You will be given 30 seconds to complete each question. After 30 seconds, the page will automatically move to the next question.

It is important that you complete this task with your own ability, so please try your best to come up with the answer on your own.

Before you start the task, let’s practice together. I will give 30 seconds to wait for you to type the answer.

Here is the question. What would be the fourth word that connects “broken – clear – eye”?

Please type your response in 30 seconds.

[The leader waits 30 seconds] What is your answer?

[If the answer is correct]: Nice try. Your answer is correct.

[If the answer is incorrect]: Nice try. The correct answer is "glass" (broken glass, clear glass, eyeglass).
[If the participant did not provide any answer]: Seems like you needed more time. Nice try. The correct answer is "glass" (broken glass, clear glass, eyeglass).

Do you have any questions?

[The leader answers the questions]

[If participants do not have further questions]: Great. Let's start.

Please complete the task in the following link: [Link to the RAT Task]

Let me know in this chat when you are finished with the task.

[After participants lets the leader know that they are finished]: Thanks. Our interaction ends here. I will now tell the experimenter to give you the further instructions.
