# Supplementary material for: The power of emojis: The impact of a leader’s use of positive emojis on members’ creativity during computer-mediated communications
Source: PLoS One. 2023 May 18;18(5):e0285368. doi: 10.1371/journal.pone.0285368 (PMC10194970; doi:10.1371/journal.pone.0285368)
Supplement: S2 Appendix — (PDF) [file pone.0285368.s003.pdf]

S2 Appendix. Information about the Emojis Used in Studies Based on Emoji Sentiment Ranking

The three emojis used across the two studies are 😃, 😊, and 👍. According to the emoji sentiment ranking,[1] all three emojis have a relatively low negativity score (p_-), a high positivity score (p_+), and a high sentiment score ($\bar{s}$), providing evidence that the emojis used in the experiments are perceived positively. Specifically, 😃 has a negativity score of .072, a positivity score of .629, and a sentiment score of .557. 😊 has a negativity score of .060, a positivity score of .704, and a sentiment score of .644. 👍 has a negativity score of .115, a positivity score of .637, and a sentiment score of .521. These scores indicate that the emojis used in our studies are generally perceived positively based on the emoji sentiment ranking.

1. Kralj Novak P, Smailović J, Sluban B, Mozetič I. Sentiment of emojis. PLoS One. 2015 10(12): e0144296. https://doi.org/10.1371/journal.pone.0144296
