# Supplementary material for: The power of emojis: The impact of a leader’s use of positive emojis on members’ creativity during computer-mediated communications
Source: PLoS One. 2023 May 18;18(5):e0285368. doi: 10.1371/journal.pone.0285368 (PMC10194970; doi:10.1371/journal.pone.0285368)
Supplement: S3 Appendix — (PDF) [file pone.0285368.s004.pdf]

S4 Appendix. Study 1 Results Controlling for Confederate Leader, Participant Age, Gender, Ethnicity, and Previous Work Experience

One-way ANOVA with Condition (0 = Control, 1 = Emoji) as the independent variable and creativity (i.e., performance on the RAT) as the dependent variable revealed that participants in the Emoji condition (*M* = 5.63; *SD* = 3.19) scored higher in terms of RAT performance than did those in the Control condition (*M* = 4.54; *SD* = 2.91), when we controlled for who was the leader among five confederates $F(1, 145) = 4.64$, $p = .033$, $\eta_p^2 = .031$, and for participants' age, $F(1, 145) = 4.59$, $p = .034$, $\eta_p^2 = .031$, gender, F(1, 145) = 4.59, p = .034, $\eta_p^2 = .031$, ethnicity, $F(1, 145) = 4.79$, $p = .030$, $\eta_p^2 = .032$, and previous work experience $F(1, 145) = 5.13$, $p = .025$, $\eta_p^2 = .034$.
