# Supplementary material for: The power of emojis: The impact of a leader’s use of positive emojis on members’ creativity during computer-mediated communications
Source: PLoS One. 2023 May 18;18(5):e0285368. doi: 10.1371/journal.pone.0285368 (PMC10194970; doi:10.1371/journal.pone.0285368)
Supplement: S4 Appendix — (PDF) [file pone.0285368.s005.pdf]

S5 Appendix. Study 1 Results Including All Participants

We conducted a one-way ANOVA with Condition (0 = Control, 1 = Emoji) as the independent variable and the creativity score as the dependent variable with all 156 participants who completed the surveys. The results show a marginally significant effect that the RAT score of the participants in the Emoji Condition ($M = 5.44$; $SD = 3.16$) was higher than the score of the participants in the Control Condition ($M = 4.51$; $SD = 2.91$), $F(1, 185) = 3.70$, $p = .056$, $\eta_p^2 = .020$.
