# Supplementary material for: The power of emojis: The impact of a leader’s use of positive emojis on members’ creativity during computer-mediated communications
Source: PLoS One. 2023 May 18;18(5):e0285368. doi: 10.1371/journal.pone.0285368 (PMC10194970; doi:10.1371/journal.pone.0285368)

S6 Appendix. Study 2 Stimuli Used in Each Condition

Control Condition

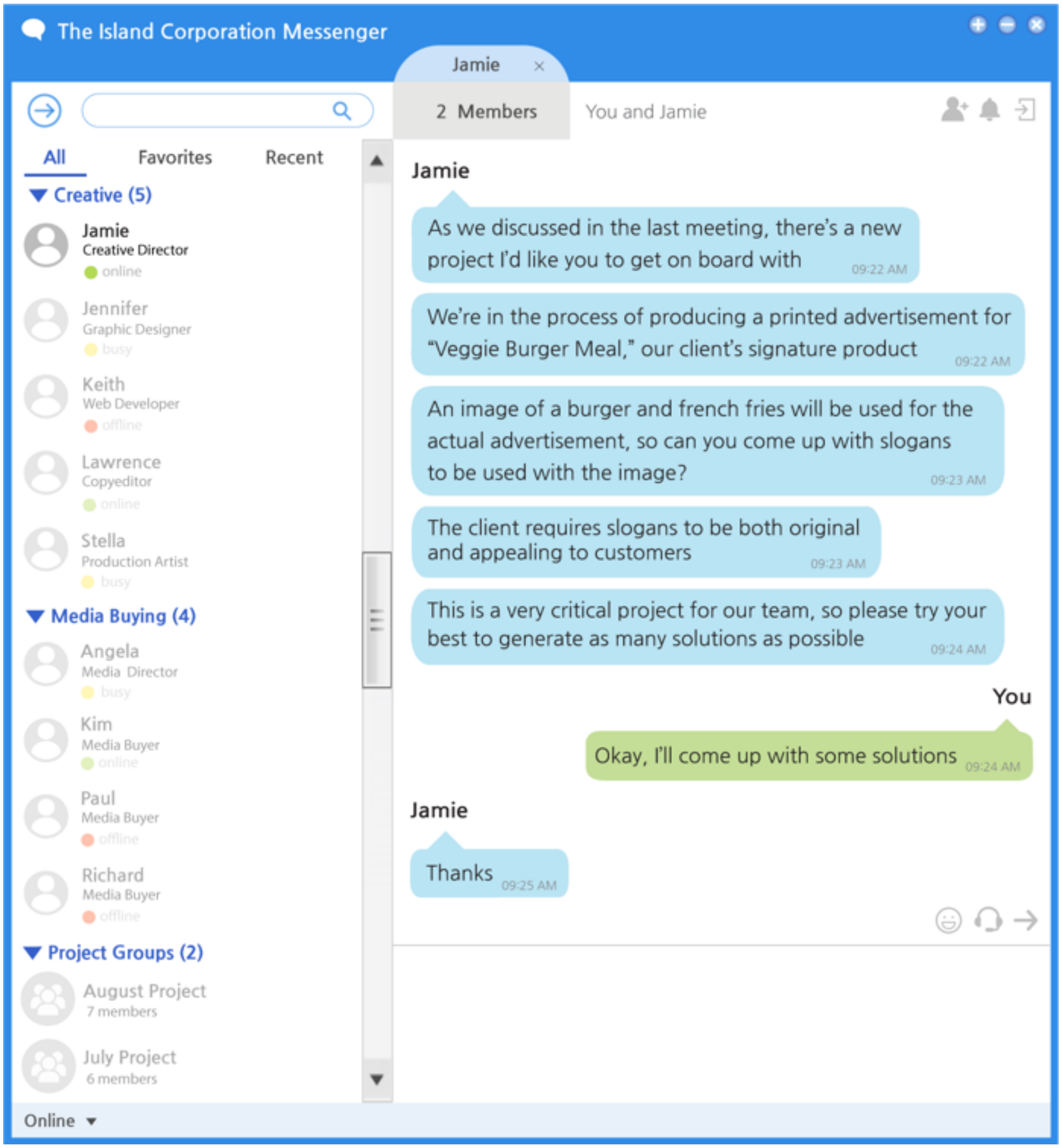

Emoji Condition

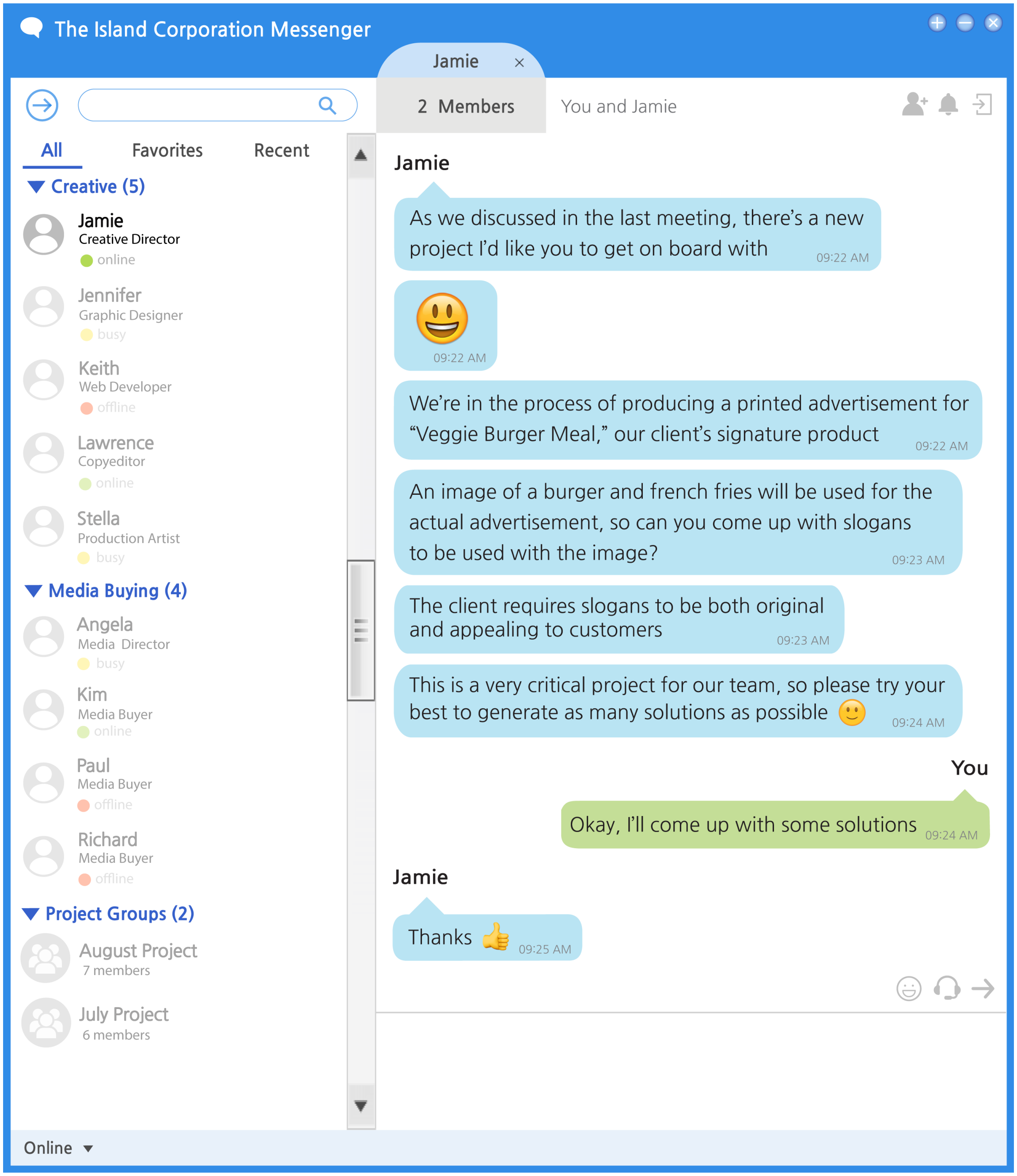

Supplement: S5 Appendix — (PDF) [file pone.0285368.s006.pdf]
