# Supplementary material for: The power of emojis: The impact of a leader’s use of positive emojis on members’ creativity during computer-mediated communications
Source: PLoS One. 2023 May 18;18(5):e0285368. doi: 10.1371/journal.pone.0285368 (PMC10194970; doi:10.1371/journal.pone.0285368)
Supplement: S6 Appendix — (PDF) [file pone.0285368.s007.pdf]

S7 Appendix. Study 2 ANOVA Results Controlling for Participant Age, Gender, Ethnicity, and Occupation

One-way ANOVA with Condition (0 = Control, 1 = Emoji) as the independent variable and creativity score as the dependent variable revealed that participants in the Emoji condition (M = 9.92; SD = 4.44) generated advertisement slogans that were significantly more creative than the slogans generated by the participants in the Control condition (M = 8.40; SD = 3.65), when we controlled for participants' age, $F(1, 157) = 5.71$, $p = .018$, $\eta_p^2 = .035$, gender, $F(1, 157) = 5.46$, $p = .021$, $\eta_p^2 = .034$, ethnicity, $F(1, 157) = 6.14$, $p = .014$, $\eta_p^2 = .038$, and occupation $F(1, 157) = 5.57$, $p = .020$, $\eta_p^2 = .034$.
