# Supplementary material for: The power of emojis: The impact of a leader’s use of positive emojis on members’ creativity during computer-mediated communications
Source: PLoS One. 2023 May 18;18(5):e0285368. doi: 10.1371/journal.pone.0285368 (PMC10194970; doi:10.1371/journal.pone.0285368)
Supplement: S7 Appendix — (PDF) [file pone.0285368.s008.pdf]

S8 Appendix. Study 2 Mediation Analyses Controlling for Participant Age, Gender, Ethnicity, and Occupation

We conducted a mediation analysis with Condition as the independent variable, perceived objectification by the leader as the mediating variable, creativity as the dependent variable, and participant age, gender, ethnicity, and occupation entered separately as the control variable in PROCESS Model 4 (5,000 bootstrap samples) [1]. The results revealed that there was a significant and positive indirect effect of the leader's use of positive emojis on members' creativity through a decrease in perceived objectification by the leader after controlling for participant age, B = .23, SE = .17, 90% CI [.01, .57], gender, B = .26, SE = .18, 90% CI [.02, .59], ethnicity, B = .24, SE = .17, 90% CI [.02, .58], and occupation, B = .24, SE = .18, 90% CI [.008, .56].
