# Supplementary material for: The power of emojis: The impact of a leader’s use of positive emojis on members’ creativity during computer-mediated communications
Source: PLoS One. 2023 May 18;18(5):e0285368. doi: 10.1371/journal.pone.0285368 (PMC10194970; doi:10.1371/journal.pone.0285368)
Supplement: S8 Appendix — (PDF) [file pone.0285368.s009.pdf]

S9 Appendix. Study 2 Moderated Mediation Analyses Controlling for Participant Age, Gender, Ethnicity, and Occupation

We conducted a moderated mediation analysis with Condition as the independent variable, the participants' relationship orientation as the moderating variable, perceived objectification by the leader as the mediating variable, creativity as the dependent variable, and participant age, gender, ethnicity, and occupation entered separately as the control variable in PROCESS Model 7 (5,000 bootstrap samples) [1].

Moderated mediation analysis controlling for participant age showed that the interaction between Condition and relationship orientation significantly predicted creativity through perceived objectification by the leader, B = .32. SE = .18, 95% CI [.03, .70]. The follow-up analyses on conditional indirect effects revealed that the positive indirect effect of the leader's use of emojis on creativity through a perceived decrease in objectification by the leader was held among individuals with a high level of relationship orientation (+1SD), B = .65, SE = .31, 95% CI [.09, 1.30], but not among those with a low level of relationship orientation (-1SD), B = -.10, SE = .21, 95% CI [-.55, .34] after controlling for participant age.

Moderated mediation analysis controlling for gender showed that the interaction between Condition and relationship orientation significantly predicted creativity through perceived objectification by the leader, B = .34. SE = .17, 95% CI [.03, .70]. The follow-up analyses on conditional indirect effects revealed that the positive indirect effect of the leader's use of emojis on creativity through a perceived decrease in objectification by the leader was held among individuals with a high level of relationship orientation (+1SD), B = .69, SE = .32, 95% CI [.12, 1.21], but not among those with a low level of relationship orientation (-1SD), B = -.10, SE = .22, 95% CI [-.54, .39] after controlling for participant gender.

Moderated mediation analysis controlling for ethnicity showed that the interaction between Condition and relationship orientation significantly predicted creativity through perceived objectification by the leader, B = .29. SE = .16, 95% CI [.007, .66]. The follow-up analyses on conditional indirect effects revealed that the positive indirect effect of the leader's use of emojis on creativity through a perceived decrease in objectification by the leader was held among individuals with a high level of relationship orientation (+1SD), B = .62, SE = .32, 95% CI [.03, 1.31], but not among those with a low level of relationship orientation (-1SD), B = -.07, SE = .19, 95% CI [-.45, .35] after controlling for participant ethnicity.

Moderated mediation analysis controlling for occupation showed that the interaction between Condition and relationship orientation significantly predicted creativity through perceived objectification by the leader, B = .32. SE = .18, 95% CI [.01, .71]. The follow-up analyses on conditional indirect effects revealed that the positive indirect effect of the leader's use of emojis on creativity through a perceived decrease in objectification by the leader was held among individuals with a high level of relationship orientation (+1SD), B = .66, SE = .32, 95% CI [.05, 1.33], but not among those with a low level of relationship orientation (-1SD), B = -.10, SE = .21, 95% CI [-.54, .34] after controlling for participant occupation.

1. Hayes A. Introduction to mediation, moderation, and conditional process analysis: A regression-based approach. New York: Guilford Press; 2013.
