# Supplementary material for: The power of emojis: The impact of a leader’s use of positive emojis on members’ creativity during computer-mediated communications
Source: PLoS One. 2023 May 18;18(5):e0285368. doi: 10.1371/journal.pone.0285368 (PMC10194970; doi:10.1371/journal.pone.0285368)
Supplement: S9 Appendix — (PDF) [file pone.0285368.s010.pdf]

S10 Appendix. Study 2 Results Including All Participants

For Study 2, we first conducted a one-way ANOVA with Condition (0 = Control, 1 = Emoji) as the independent variable and the creativity score as the dependent variable with all 187 participants. The results show a marginally significant effect that participants in the Emoji Condition (M = 9.44; SD = 4.55) generated advertising slogans that were more creative than the slogans generated by participants in the Control Condition (M = 8.28; SD = 3.67), $F(1, 185) = 3.70$, $p = .056$, $\eta_p^2 = .020$.

Then, we conducted a moderated mediation analysis with Condition as the independent variable, participant's relationship orientation as the moderating variable, perceived objectification by the leader as the mediating variable, creativity as the dependent variable using PROCESS Model 7 (5,000 bootstrap samples). The results show that the interaction between Condition and relationship orientation significantly predicted creativity through perceived objectification by the leader, B = .50. SE = .21, 95% CI [.14, .97]. The follow-up analyses on conditional indirect effects revealed that the positive indirect effect of the leader's use of emojis on creativity through a perceived decrease in objectification by the leader was held among individuals with a high level of relationship orientation (+1SD), B = .89, SE = .32, 95% CI [.32, 1.58], but not among those with a low level of relationship orientation (-1SD), B = -.43, SE = .31, 95% CI [-1.13, .10]. The results on all participants are consistent with our findings on participants who passed the manipulation check and attention check.

1. Hayes A. Introduction to mediation, moderation, and conditional process analysis: A regression-based approach. New York: Guilford Press; 2013.
